# Intense light unleashes male–male courtship behaviour in wild-type *Drosophila*

**DOI:** 10.1098/rsob.220233

**Published:** 2023-07-19

**Authors:** Atsushi Ueda, Abigayle Berg, Tashmit Khan, Madeleine Ruzicka, Shuwen Li, Ellyn Cramer, Atulya Iyengar, Chun-Fang Wu

**Affiliations:** ^1^ Department of Biology, University of Iowa, Iowa City, IA 52242, USA; ^2^ Iowa Neuroscience Institute, University of Iowa, Iowa City, IA 52242, USA; ^3^ Department of Biological Sciences, University of Alabama, Tuscaloosa, AL 35487, USA

**Keywords:** male–male courtship, ethology, automated locomotion tracking, sensory-motor processing, *Drosophila* mutants

## Abstract

*Drosophila* courtship studies have elucidated several principles of the neurogenetic organization of complex behaviour. Through an integration across sensory modalities, males perform stereotypic patterns of chasing, courtship song production and copulation attempts. Here we report a serendipitous finding that intense light not only enhances courtship toward female targets but also triggers unexpected courtship behaviours among male flies. Strikingly, in wild-type male-only chambers, we observed extreme behavioural manifestations, such as ‘chaining’ and ‘wheeling’, resembling previously reported male–male courtship behaviours in *fruitless* mutants and in transformants with ectopic *mini-white^+^* overexpression. This male–male courtship was greatly diminished in a variety of visual system mutants, including disrupted phototransduction (*norpA*), eliminated eye-colour screening pigments (*white*), or deletion of the R7 photoreceptor cells (*sevenless*). However, light-induced courtship was unhampered in wing-cut flies, despite their inability to produce courtship song, a major acoustic signal during courtship. Unexpectedly the olfactory mutants *orco* and *sbl* displayed unrestrained male–male courtship. Particularly, *orco* males attained maximum courtship scores under either dim or intense light conditions. Together, our observations support the notion that the innate male courtship behaviour is restrained by olfactory cues under normal conditions but can be unleashed by strong visual stimulation in *Drosophila*.

## Graphical abstract

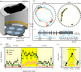

## 1. Introduction

The male courtship behaviour repertoire in *Drosophila melanogaster* emerges from a complex integration of external sensory information [1–3], prior experiences [4–6] and internal drive [7–9]. A large collection of discrete visual [10–12], olfactory [13–15], gustatory [16–18] and mechanosensory [19] cues serve to direct courtship behaviour towards receptive females. These signals in turn trigger stereotypic sequences of chasing, licking, wing extension, courtship ‘song’ production and eventual mounting [20,21]. Genetic perturbations in this sensory-motor integration process can reveal contributions of the genetic organizations and neural circuit mechanisms underlying this complex behavioural programme [22–24].

A striking phenotype arising from certain genetic manipulations is male–male courtship behaviours. For example, several *fruitless* mutants display remarkable ‘chains’ of courting males [25,26]*.* The *fruitless* gene product undergoes alternative splicing to produce female- and male-specific transcripts, *fru^F^* and *fru^M^,* respectively [27,28]. In *fru^M^-*disrupted males, male–male courtship is often observed [27]. Disruption of subsets of *fru^M^-*positive sensory neurons hampers reception of the male-specific repulsive cues, 7-tricosine and *cis*-vaccenyl acetate, leading to male–male courtship [29–33]. Furthermore, it has been reported that optogenetic activation of *fru^M^*-expressing neural circuits induces male–male courtship [34]. Male–male courtship has also been reported in studies using *painless* TrpA channel mutants [35], dopaminergic signalling mutants [36,37] or transformants for forced expression of mini-*white* constructs [38,39].

Here, we describe a serendipitous finding of male–male courtship behaviours in wild-type (WT) *Drosophila melanogaster* evoked by intense light. We observed the light intensity-dependent increase in chasing, wing extension, courtship song, and chaining in different strains of WT males, using LED, incandescent, and sunlight illumination. We examined the phenomena in flies with genetic and surgical manipulations to elucidate the key roles of the visual and olfactory systems in gating this male–male courtship behaviour.

## 2. Results

### 2.1. Intense light triggers courtship behaviour in male flies

**Figure 1.**
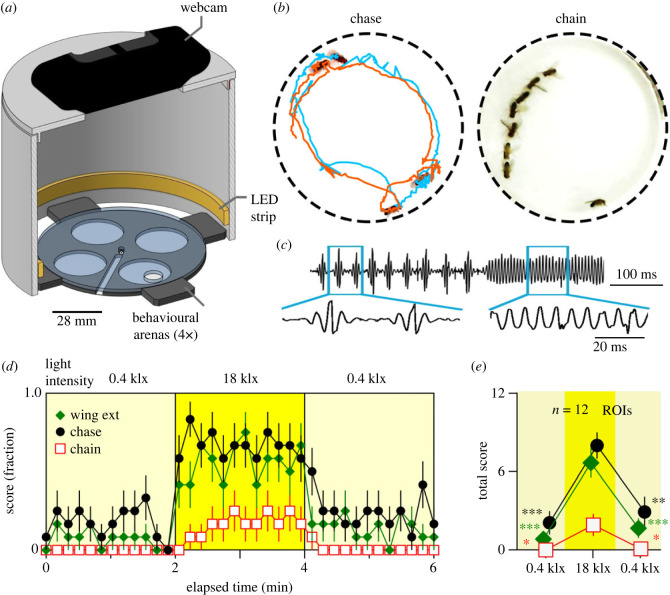
Light-evoked male–male courtship behaviour in *Drosophila*. (*a*) Schematic drawing of the behavioural arena, LED strip lighting and webcam configuration. Eight males were loaded into each of the four circular behavioural arenas (regions of interest, ROIs). An LED strip surrounding the arenas illuminated fly behaviour with minimal shadowing, and a webcam above recorded activity. (*b*) Intense light triggers male–male courtship behaviours including chase (left, orange track chasing cyan track), as well as chain and wing extensions (right). (*c*) In a modified arena equipped with a microphone (see Methods), intense light triggered pulse and sine songs among male flies, resembling qualitatively songs recorded during male–female courtship (electronic supplementary material, figure S1). (*d*) Quantification of light-induced male–male courtship behaviours. Eight males were subjected to a 2-min low-light intensity period, followed by a 2-min high-intensity light, and a 2-min low-light recovery period. For each successive time bin (10 s), the presence or absence of respective behaviours was scored (1 or 0). Data points presented show the average score pooling from 12 ROIs for the various behaviours (see Methods). Error bars indicate SEM. (*e*) Total scores in (*d*) are summed over 2-min periods and shown with SEM and ROIs. One-way ANOVA, Bonferroni-corrected *t*-test *post hoc* was applied for comparisons between first low-light and high-light, as well as between high-light and the second low-light. *p < 0.05, **p < 0.01, ***p < 0.001.

A modified semi-automated *Drosophila* tracking system, IowaFLI Tracker [40], was employed to analyse activity in each circular arena containing eight flies (figure 1). The tracking system consisted of a clear polyacrylic sheet containing four arenas which were housed in a light-shielded cylindrical chamber equipped with an inner circular strip of LED lighting (figure 1*a*). A webcam mounted on top of the ceiling captured fly behaviours. In male-only arenas, we unexpectedly discovered a high frequency of courtship behaviours at intense light setting. Seconds after light on, we regularly observed chasing and attempting to court among male flies. Strikingly, we often saw chains of courting males (figure 1*b*; electronic supplementary material, movie S1), reminiscent of phenotypes previously found in *mini-white* transgenic lines [38,39] and *fruitless* mutants [25,41]. To quantify light-induced courtship, we developed a protocol where the eight flies in the arena were first subjected to 2-min illumination of relatively low intensity (0.4 klx, within the normal range of room lighting) followed by 2 min of high-intensity light (18 klx) and subsequently, another 2-min low-intensity light period (figure 1*d*). For each successive 10-s interval, we manually scored the occurrence (0 or 1) of chasing, wing extensions and chaining in the arena. The total number of intervals during which the respective behaviours were observed in the arena was reported as the ‘total score’ (ranging from 0 to 12 for the 2-min period, figure 1*d*,*e*; see Methods). During the high-light period, the increase in total chasing score was more than 700%, approximately 400% for wing extension, and greater than 800% for chasing (figure 1*e*).

In a modified arena equipped with a microphone, we confirmed the light-induced wing extension behaviour was related to song production among male flies (figure 1*c*; electronic supplementary material, figure S1). We found that like male–female courtship songs, male–male songs consisted of pulse and sine songs. The frequency and duration of sine songs as well as the inter-pulse intervals and number of pulses per bout of the pulse song were analysed, showing overlapping ranges of these parameters between male–male and male–female songs (electronic supplementary material, figure S1*b*).

### 2.2. Generality and specificity of light-induced courtship behaviours

**Figure 2.**
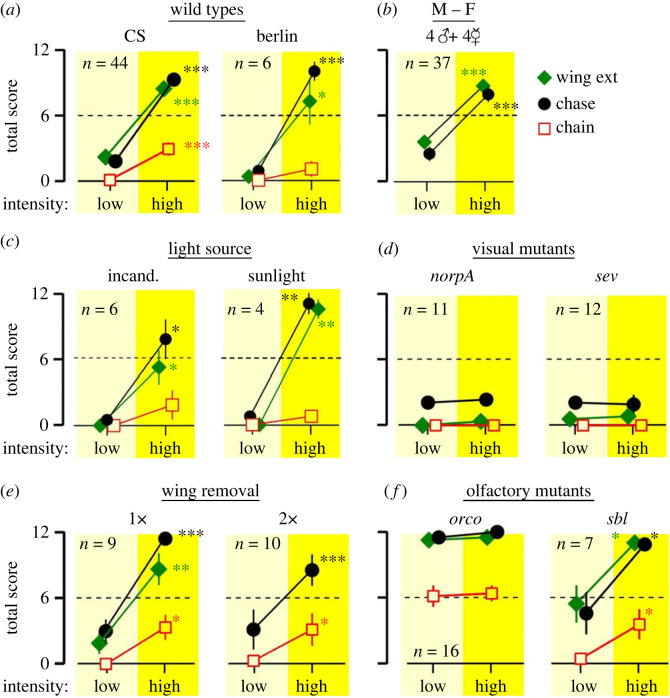
Genetic and environmental factors affecting light-evoked male–male courtship. Flies were subjected to a 2-min period of low light intensity followed by 2 min of high-intensity illumination (0.4 klx and 18 klx respectively, except for (*c*) with different light sources). (*a*) Light-evoked male–male courtship in the CS and Berlin WT *Drosophila* strains. (*b*) Courtship behaviour in mixed sex arenas with 4 males and 4 females. (*c*) Male–male courtship in arenas under incandescent light (left) or in direct sunlight (right). For incandescent illumination, light was delivered by two light pipes from an USHIO EKZ 10.8 V, 30 W bulb (‘low’ intensity: 0.4 klx; ‘high’ intensity: 25 klx). For sunlight experiments, the behavioural arena was placed in a shaded area for the ‘low’ period (approx. 0.4 klx) and moved to direct sunlight (approx. 90 klx) for ‘high’ period. Experiments were done in the Biology Building courtyard on the University of Iowa campus on 19 May 2022 at 15.00 CDT (temperature approx. 29°C). (*d*) Lack of light-triggered courtship behaviours in visual mutants (*norpA* and *sev*). (*e*) Wing-cut flies and (*f*) olfactory mutants (*orco* and *sbl*). Note the light-induced increase in courtship behaviour is absent in visual mutants, but unimpaired in flies with one (1x) or both (2x) wings removed. Olfactory mutants show greatly enhanced courtship activity, with *orco* attaining nearly maximum scores even at low light intensity. Data are shown as mean ± SEM. *p < 0.05, **p < 0.01, ***p < 0.001, paired *t*-test (high versus low). For each panel, the sample size (ROIs) is indicated.

To determine whether light-induced male–male courtship is a specific phenomenon restricted to our WT *Canton-S* (CS) strain used in most of the experiments, we tested another WT strain of *melanogaster*, *Berlin*, and found the same male–male courtship behaviours as shown in figure 2*a*. We observed similar high-light-induced increases in wing extension and chasing behaviour as in CS flies, with occasional chaining observed as well (figure 2*a*). We also observed mixed-sex arenas (4 male and 4 female flies) to examine how high-intensity illumination affects male–female interactions, to determine whether the intense light-triggered courtship behaviour was restricted towards other males only. As shown in figure 2*b*, in these mixed-sex arenas, high-intensity lighting also similarly increased male–female courtship behaviours (approx. 300%). In a subset of mixed-sex arenas, we determined the kinetics of intense-light effect on male–female courtship scores and found temporal properties similar to male-only arenas (electronic supplementary material, figure S2). Together, these findings suggest intense light intensifies courtship drive of male flies towards both male and female flies, rather than switches their sex preference from females to males.

### 2.3. Roles of sensory cues in light-triggered male–male courtship

We characterized the efficacy of different lighting conditions for triggering the male–male phenomenon. By adjusting the arena LED light intensity, we found that the relationship between light intensity and incidence of wing extensions, chasing and chaining behaviours was monotonic and that the proportionally steepest increases occurred between 2.0 and 6.0 klx (electronic supplementary material, figure S3). We then asked whether the LED light source we used was specifically required, or some other sources could also evoke the effect. As shown in figure 2*c*, intense incandescent light (25 klx) also elicited courtship behaviour. We further asked if natural light (e.g. sunlight on a spring day) could induce male–male courtship activity. Remarkably, after moving the arena from a shaded location (approx. 0.4 klx) to a location under direct bright sunlight (approx. 90 klx), we observed robust courtship activity (figure 2*c*). These results indicate that a wide variety of sufficiently intense, broad-spectrum visible light sources (see electronic supplementary material, figure S4, for spectral measurements of different light sources) can effectively induce male–male courtship behaviour.

We introduced experimental perturbations to the various sensory systems for indications of their roles in the light-induced male–male courtship. First, we examined males with a disrupted visual system. The gene *norpA* encodes the enzyme phospholipase C required for phototransduction, and several mutations eliminate photoreceptor potential, rendering mutant flies completely blind [42]. In blind *norpA^P12^* mutants, we found intense light did not induce courtship activity (figure 2*d*; electronic supplementary material, movie S1).

We further took advantage of anatomical mutations affecting a particular category of photoreceptors. The *Drosophila* compound eye is composed of about 800 facets, each cylindrical rhabdomere below consisting of 6 outer photoreceptors (R_1_–R_6_, green light sensitive) and two central photoreceptors (R_7_ and R_8_, UV and blue light, respectively). In *sevenless* (*sev*) mutants, the high-acuity channels composed of central R_7_ and R_8_ are functionally impaired because R_7_ is absent, which also disrupts the light guide for R_8_ underneath, while the high sensitivity channel, consisting of R_1_–R_6_, is intact [43]. The results showed that light-evoked male–male courtship was absent in *sev* mutants (figure 2*d*), implying a critical role for the R_7_/R_8_ system in light-triggered male–male courtship. Visual processing depends on integration of photoreceptor responses from individual rhabdomeres. Because of the screening pigments, each rhabdomere acts as an independent light guide for input from a narrow angular visual field, in isolation of its neighbours [44]. Mutations of the *white* (*w*) gene are devoid of the screening pigments and thus degrade visual image processing, in spite of increased sensitivity to light [45]. In *w* mutant alleles, including *w^1118^* and *w^G^*, despite their active locomotion in the arena, the male–male courtship behaviour was missing (electronic supplementary material, figure S5). Conceivably, visual acuity is crucial to the locomotion control during courtship. It is documented that *white* mutant males show lower performance in male–female courtship [46,47]. Further, additional eye colour mutants have been reported to be defective in courtship behaviour [48]. We have examined *cinnabar* (*cn*) and *cinnabar brown* (*cn bw*). The *cn* flies lack the screening pigment ommochrome [49] and show bright orange eye colour; *bw* lack pteridine [49] and *cn bw* flies are white-eyed. Our initial observations suggest that both *cn* and *cn bw* mutants showed greatly decreased intense-light-induced male–male courtship, comparable to the *w* alleles described above. These observations indicate the central role of visual function in the light-evoked male–male courtship.

By contrast to the visual system, manipulations of mechanosensory or auditory cues revealed drastically different outcomes. In groups of males, courtship behaviours are known to be triggered by playback of courtship song [19]. However, we found that in wing-cut male flies, which had disrupted wing mechanosensory inputs and were unable to produce courtship song, intense light nevertheless reliably triggered chasing and chaining (figure 2*e*). Notably, flies with one wing or both wings cut displayed similar chasing and chaining scores and flies with only a single wing could attain a frequency of wing extension comparable to WT. This result demonstrates that the auditory cues generated by wing beats and the mechanosensory receptors along the wing blades are not required in the light-evoked male–male courtship.

To determine the role of olfactory systems, we examined two mutants: *smellblind* (*sbl*), a *para* Na^+^ channel allele with disrupted odour-evoked behaviours [50], and *odorant receptor co-receptor* (*orco*), encoding a requisite olfactory receptor subunit [51]. Surprisingly, at low light intensities, both mutants displayed strikingly elevated courtship behaviours compared to WT males (figure 2*f*). Particularly, recurrent male–male courtship in *orco* flies was so prevalent (electronic supplementary material, movie S1), frequently approaching the maximum score of 12, that a further increase in courtship was not apparent during the intense light periods. However, in *sbl* males, which showed milder increases in low-light scores, intense light could further promote courtship activities to attain the maximum score (figure 2*f*), prompting a further investigation into interactions between intense light and olfactory processing in gating male courtship behaviours.

Apparently, olfactory processing plays a similar role in both male–male and male–female courtship behaviours (electronic supplementary material, figure S6). We monitored the intense-light effect on male–female courtship in *sbl* and *orco* mutant flies. Consistent with male–male courtship, *sbl* male–female courtship behaviour was further increased by intense light, while a less obvious effect on *orco* was observed because their chasing and wing extension scores were already near saturation at the low light intensity.

### 2.4. Spatio-temporal properties of male–male courtship behaviours

**Figure 3.**
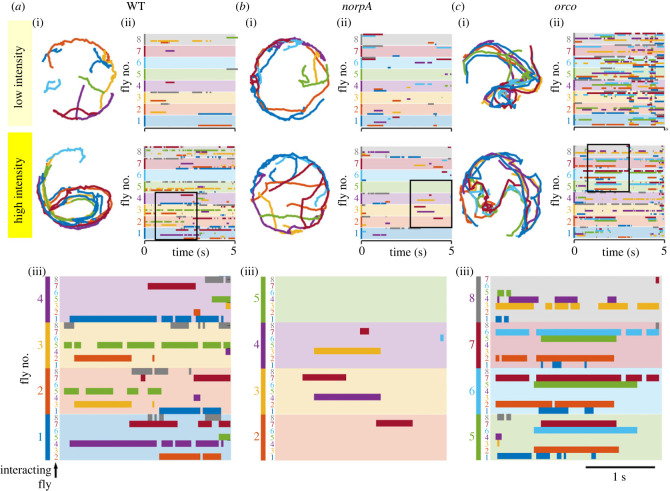
Fly locomotion tracks and interactograms of light-induced male–male courtship. Representative behavioural trajectories (i; left panels for each genotype; 5-s sample) and corresponding interactograms (ii; right panels) of (*a*) WT, (*b*) *norpA*, and (*c*) *orco* males under low and high light. Note that flies are chasing others under high light in WT, and under both low and high light in *orco* mutants. However, *norpA* flies displayed no increase in levels of locomotion or social interactions. To monitor fly activity patterns, the IowaFLI Tracker [40] was used for automated analysis of kinematic properties of individual flies as well as social interactions among them. An interaction proximity criterion of 3.75 mm, optimal for capturing chasing events, was adopted. For each genotype, individual flies (1–8) were colour-coded to depict their continuous locomotion tracks (i), and to register events of their interactions with others in the interactogram (each colour-coded, falling within the 3.75-mm mutual distance) along the 5-s duration (ii). In the horizontal shaded bands of 8 different colours, interactions of the fly of the designated band colour with all other individuals were registered with darker colour-coded line segments to indicate the time intervals when their mutual distance less than 3.75 mm. (iii) A selected area (boxed) in the interactogram is enlarged to better illustrate the reciprocal relationship among the interacting individuals identifiable with assigned colours.

In addition to courtship event statistics shown in figure 2, we also monitored continuous activity patterns among the male flies, using IowaFLI Tracker [40] for an automated analysis of kinematic properties as well as social interactions among individually identified flies. As figure 3*a* shows, WT flies, upon high-intensity illumination, displayed a drastic increase in activity levels (panel (i), locomotion tracks, with individual flies colour-coded) as well as a greatly enhanced level of social interactions (panels (ii), interactograms, with coloured time segments registering other individuals active within the 3.75 mm vicinity; an interaction proximity criterion of 3.75 mm between centroids was adopted because it optimally captures chasing events in WT flies). By contrast, *norpA* flies, upon high-intensity illumination, displayed neither a drastic increase in activity levels (panel (i)) nor a greatly enhanced level of social interactions (panel (ii)). No male–male chasing or wing extension events were evoked by intense light (figure 2*d*). Interestingly, *orco* flies exhibited a contrasting case for reduced response to intense light. Chasing or chaining events already occurred at low light intensities, indistinguishable from behavioural activities under intense light, as indicated by the locomotion tracks (panel (i)) and interactograms (panel (ii)).

In figure 3, a selected area (boxed) in the interactogram panel for each genotype is enlarged to illustrate interaction records for individually identified flies (panel (iii)). Each fly in the arena was assigned a number and a colour code. In each colour-shaded strip for an identified fly, the other flies in the vicinity of this individual are recorded as bar segments of darker colours. Thus, the reciprocal relationship among the colour-coded individuals can be traced for each time point in the original video record (15 frames s^−1^). The interactogram provides the raw source data for time-stamped proximity information for the involved individuals. Following visual inspection, particular categories of courtship interactions (wing extension, chasing, and chaining) could be related back to the video recording and the locomotion tracks to obtain a more comprehensive description of the event.

IowaFLI Tracker also provides statistics for kinematics parameters, such as distance travelled, speed, and % time moving (electronic supplementary material, table S1). From the table, *norpA* is clearly hyperactive (with high values of speed, distance travelled, and % time moving). However, most of the recorded *norpA* interactions, defined by the 3.75-mm proximity criterion, were not courtship-related and occurred most often along the arena periphery (figure 3*b*(i)). By contrast, many interactions indicated in the interactograms for WT and *orco* could be related to compact bundles of locomotion tracks traversing across the arena for the same time segments (figure 3*a*(i)(ii),*c*(i)(ii)).

## 3. Discussion

### 3.1. The role of visual system function in male–male courtship

Our findings add a striking case of male–male courtship behaviour in WT flies, in contrast to previous reports from a number of genetic variants, including *fru* [25,41], *painless* [35], an unidentified mutation (on Chr. 3L) reported by Sharma [52], as well as several mini-*w^+^* transformants [38,39]. A strong visual contribution to such behaviours was suggested by Sharma's observation that blue light inhibits male–male courtship [52]. Similarly, blue and UV lights have been reported to suppress male–female courtship [6]. In a preliminary follow-on experiment, we subjected *orco^1^* and *orco^2^* males to intense blue light and found that male–male courtship was severely suppressed (electronic supplementary material, table S2). It is important to note that the two *orco* alleles employed in this study carry the mini-*w^+^* construct (in place of the *orco* gene) in a *w^−^* background (see Methods for the construct). Their eye colour appeared normal, indicating strong mini-*w^+^* expression. Therefore, the extreme phenotypes of *w*;;*orco^1^* and *w*;;*orco^2^* in this report may have had contributions from mini-*w^+^* expression.

Our preliminary analysis of *orco^1^* and *orco^2^* heterozygotes (*orco*/+) generated from crossing *w*/Y; *orco* males to *w^1118^* females displayed a correlation between eye-colour density and male–male courtship activity. We observed darker eye colour in *w*;;*orco^1^*/+ than *w*;;*orco^2^*/+, indicating differences in mini-w^+^ expression, and accordingly a stronger male–male courtship behaviours in w;;*orco^1^*/+ (electronic supplementary material, table S3). In terms of male–male courtship scores, we observed in the following sequence as shown in electronic supplementary material, table S3:

$$w;orco^{1}/orco^{1}≃w;orco^{2}/orco^{2}>w;orco^{1}/+>w;orco^{2}/+.$$

Furthermore, *norpA^P12^*/Y;;*orco^1^*/+ and *sev^LY3^*/Y;;*orco^1^*/+ males have been observed to behave like *norpA^P12^* and *sev^LY3^* males, suggesting total suppression of the *orco^1^*/+ phenotype by the visual mutations, (electronic supplementary material, table S3). Taken together, our results confirm a critical role of visual system function in male-male courtship.

### 3.2. The effective light intensity range and underlying candidate neural circuits

The above findings demonstrate that light plays a major role in courtship behaviour in *Drosophila melanogaster*. Besides facilitating visually guided motor behaviours, intense light serves as a potent modulator promoting male sex drive as indicated by increased courtship behaviour towards males as well as females. Notably, the light intensity range required to effectively trigger male–male courtship (between 2 and 6 klx; electronic supplementary material, figure S3) is several fold greater than common light sources encountered in the laboratory, including room lights or incubator lighting (approx. 0.5 klx, measurements in our laboratory). However, this light intensity is within the range that flies encounter in the wild, as direct sunlight can be above 100 klx [53]. Importantly, our findings with both incandescent light and sunlight (figure 2*c*) indicate a variety of sufficiently intense broad-band light sources (electronic supplementary material, figure S4) can induce male–male courtship.

It is tempting to speculate based on recent advances in identification and manipulation of neuronal components of various circuits involved in male courtship behaviour. Our analysis suggests an interplay of visual and olfactory processing. Indeed, in a model previously proposed by Wang *et al*. [35], male flies have an intrinsic drive to court that is normally restrained by olfactory processing of certain signals from other males (e.g. *cis*-vaccenyl acetate [22]). Flies deficient in olfactory processing such as *orco* and *sbl* would lack these restraints, consistent with the increased male–male courtship shown in figures 2 and 3. Conceivably, intense light could serve to unleash the intrinsic courtship drive either by supercharging the courtship motor programme to overcome olfactory inhibition or by directly suppressing the inhibitory olfactory processing in WT flies.

Within the broad-band illumination used in this study, the shorter wavelength UV and blue light preferentially activate R_7_ and R_8_, respectively. UV and blue light are known to suppress male–female courtship [6] and blue light was found to inhibit male–male courtship in the mutant flies reported by Sharma [52]. Our analysis of *norpA* and *sev* mutants implies that R_7_ and/or R_8_ processing plays an important role in light-induced courtship (figure 2*d*). Photoreceptors R_7_ and R_8_ directly target several classes of medulla neurons, including Dm8 amacrine-like cells, and Tm5 and Tm9 cells which project to the lobula plate [54]. It is possible that these medulla neurons directly or indirectly influence excitability of *fru-*expressing P1 neurons in the lobula plate, which exert strong modulation on the activity of LC10a circuit that has been shown to be essential for controlling courtship chasing performance [3,55]. Future analysis will elucidate the action and modulation of the neural circuit underlying light-evoked male–male courtship. Our findings provide a new context of courtship behaviour and can add constraints for data interpretation in the studies of neural circuits responsible for the repertoires in male courtship behaviours.

## 4. Methods

### 4.1. *Drosophila* stocks and husbandry

All flies were reared at room temperature (23°C) under a 12:12 light–dark cycle in vials containing cornmeal media [56,57]. All flies studied were age-controlled as specified. Throughout, the study WT strain used was *Canton-S* (except for figure 2*a*, where WT-Berlin was used, gift from M. Heisenberg [44]). Other mutant lines studied included: *smellblind* (*sbl^1^* and *sbl^2^*), EMS-induced mutant alleles of the voltage-gated sodium channel gene *paralytic* (gift from J. Carlson [50]); *orco^1^* and *orco^2^*, two insertion alleles of the gene *odorant receptor co-receptor* (Bloomington 23129 [w*; TI{w+mW.hs=TI}*Orco^1^*] and 23130 [w*; TI{w+mW.hs=TI}*Orco^2^*] respectively); *norpA^P12^*, an EMS-induced allele of *no receptor potential A*, encoding a phospholipase C required for visual transduction (gift from W. L. Pak [42]); *sevenless^LY3^* (*sev^LY3^*), lacking R7 photoreceptors (gift from W. L. Pak [58]); *cn^1^* (Bloomington 263); and two alleles of *white*, *w^1118^* (Bloomington 6326) and *w^G^* (gift from M. L. Joiner [59]).

### 4.2. Courtship behaviour recording

Fly courtship behaviour was observed in a custom-built chamber which consisted of an arena piece, and arena ceiling cover, and a light-shielded cylindrical chamber (figure 1*a*). The arena piece consisted of a Plexiglas sheet (2 mm thick) with four milled circular chambers referred to as ‘regions of interest’ or ROIs (26 mm in diameter) seated on filter paper (Whatman No. 1) forming 2-mm-high behavioural arenas (4 ROIs). The arena ceiling cover was a Plexiglas sheet of the same specification. The light-shielded chamber was constructed from a 4-inch PVC pipe coupler (NIBCO 4801) with a custom milled PVC lid. A 21 LED strip (STN-A30K80-A3A-10B5M-12 V, SuperBrightLEDs.com) was glued along the inner rim next to the base of the light chamber. LEDs were powered by a custom-built current-controlled power supply (electronic supplementary material, figure S7). For figure 2*c* experiments, incandescent light was delivered via fibre optic light pipes from an USHIO EKZ 10.8 V, 30 W bulb. Spectral characteristics of the above light sources were determined across a range of power supply currents using a flame miniature spectrometer (Ocean Optics, Orlando, FL; electronic supplementary material, figure S4*a*–*c*) or Goyalab GoSpectro (Axiom Optics, Somerville, MA; electronic supplementary material, figure S4*d*). The spectral power across a range of wavelengths (*λ* = 189 nm to 884 nm) was measured following manufacturer's instructions. In electronic supplementary material, figure S4, spectral data were normalized to the peak emission detected (i.e. *E*(*λ*)/*E*(peak) for the wavelength *λ*). In addition, the spectral characteristics of sunlight on a sunny spring day (14.00, 10 April 2023, in Tuscaloosa, AL, USA) were measured (electronic supplementary material, figure S4).

Fly behaviours were captured by a Logitech c920 webcam connected to a PC. For microphone recordings (figure 1*b*), a modified arena of a larger dimension was constructed with a hole drilled in the side of the arena. A high-gain microphone (FC-23629-P16, Knowles Electronics, Itasca, IL, USA; see also [60]) was placed in the opening to pick up fly sounds. Light intensity measurements (electronic supplementary material, figure S2) were performed using a digital light meter (DLM1, Universal Enterprise Inc., Portland, OR, USA) following manufacturer's instructions.

Behavioural recordings were performed by aspirating 8 flies into each arena (without anaesthesia). Following a brief acclimatization period (less than 5 min), flies were recorded for 2 min under ‘low-light’ conditions, immediately followed by 2 min of ‘high-light’ conditions. In some experiments (figure 1*d*,*e*), we included a 2-min ‘low-light’ recovery period.

### 4.3. Quantification and statistical analysis

Behavioural scoring was carried out in groups of 8 flies confined in the arena (for male–female courtship, 4 males and 4 females were used). Criteria for chasing and wing extension behaviours were based on Hall [20], and we defined chaining behaviour as the formation of a mobile chain of 4 or more flies (cf. Zhang & Odenwald [38] and Hing & Carlson [39]). Scoring was performed by a pair of operators (an observer and a recorder), either on-site during live video recording or by viewing playback of videos. Recording and scoring were performed on pairs of ROIs, one for experimental and one for control (or comparison) flies. Each successive 10-second interval in the 2-min observation periods was divided into two 5-second scoring windows and experimental and control ROIs were scored alternately (i.e. 5 s for one ROI followed by 5 s for the other). The presence or absence of each of the courtship behaviours in an ROI was recorded in the 5-second window. The total number of 5-second windows (out of 12 possible) in which the respective behaviours were observed were reported as the ‘total score’, ranging from 0 to 12. In addition, automated analysis of walking kinematics was performed offline with IowaFLI Tracker as described in Iyengar *et al*. [40].

All statistical analyses were performed in MATLAB r2021b. Unless otherwise indicated, all statistical significance values are reported from paired *t*-tests comparing the ‘low’ versus ‘high’ light conditions. For comparisons across multiple groups (figure 1*e*; electronic supplementary material, figure S2*d*), a one-way ANOVA was performed as a preliminary step. Electronic supplementary material, table S4, indicates the complete list of statistical analyses and results in this study.

## Data Availability

An updated version of IowaFLI Tracker (version 3.1) and the original data sets for figure and table construction can be found on github (https://github.com/IyengarAtulya/IowaFLI_tracker). Additional information is provided in electronic supplementary material [61].

## References

[1] Greenspan RJ, Ferveur J-F. 2000 Courtship in drosophila. Annu. Rev. Genet. **34**, 205. (10.1146/annurev.genet.34.1.205) 11092827

[2] Ferveur J-F. 2005 Cuticular hydrocarbons: their evolution and roles in *Drosophila* pheromonal communication. Behav. Genet. **35**, 279-295. (10.1007/s10519-005-3220-5) 15864443

[3] Clowney EJ, Iguchi S, Bussell J, Scheer E, Ruta V. 2015 Multimodal chemosensory circuits controlling male courtship in *Drosophila*. Neuron **87**, 1036-1049. (10.1016/j.neuron.2015.07.025) 26279475 PMC4560615

[4] Quinn WG, Greenspan RJ. 1984 Learning and courtship in *Drosophila*: two stories with mutants. Annu. Rev. Neurosci. **7**, 67-93. (10.1146/annurev.ne.07.030184.000435) 6143528

[5] Griffith LC, Ejima A. 2009 Courtship learning in *Drosophila melanogaster*: diverse plasticity of a reproductive behavior. Learn. Mem. **16**, 743-750. (10.1101/lm.956309) 19926779 PMC4419844

[6] Sakai T, Isono K, Tomaru M, Fukatami A, Oguma Y. 2002 Light wavelength dependency of mating activity in the *Drosophila melanogaster* species subgroup. Genes Genet. Syst. **77**, 187-195. (10.1266/ggs.77.187) 12207040

[7] Zhang SX, Rogulja D, Crickmore MA. 2019 Recurrent circuitry sustains *Drosophila* courtship drive while priming itself for satiety. Curr. Biol. **29**, 3216-3228.e9. (10.1016/j.cub.2019.08.015) 31474539 PMC6783369

[8] Liu W, Ganguly A, Huang J, Wang Y, Ni JD, Gurav AS, Aguilar MA, Montell C. 2019 Neuropeptide F regulates courtship in *Drosophila* through a male-specific neuronal circuit. eLife **8**, e49574. (10.7554/eLife.49574) 31403399 PMC6721794

[9] Terhzaz S, Rosay P, Goodwin SF, Veenstra JA. 2007 The neuropeptide SIFamide modulates sexual behavior in *Drosophila*. Biochem. Biophys. Res. Commun. **352**, 305-310. (10.1016/j.bbrc.2006.11.030) 17126293

[10] Agrawal S, Dickinson MH. 2019 The effects of target contrast on *Drosophila* courtship. J. Exp. Biol. **222**, jeb203414. (10.1242/jeb.203414) 31315932

[11] Cook R. 1980 The extent of visual control in the courtship tracking of *D. melanogaster*. Biol. Cybernetics **37**, 41-51. (10.1007/BF00347641)

[12] Kimura K, Sato C, Yamamoto K, Yamamoto D. 2015 From the back or front: the courtship position is a matter of smell and sight in *Drosophila melanogaster* males. J. Neurogenet. **29**, 18-22. (10.3109/01677063.2014.968278) 25257899

[13] Datta SR et al. 2008 The *Drosophila* pheromone cVA activates a sexually dimorphic neural circuit. Nature **452**, 473-477. (10.1038/nature06808) 18305480

[14] Gailey DA, Lacaillade RC, Hall JC. 1986 Chemosensory elements of courtship in normal and mutant, olfaction-deficient *Drosophila melanogaster*. Behav. Genet. **16**, 375-405. (10.1007/BF01071319) 3092798

[15] Grosjean Y, Rytz R, Farine J-P, Abuin L, Cortot J, Jefferis GSXE, Benton R. 2011 An olfactory receptor for food-derived odours promotes male courtship in *Drosophila*. Nature **478**, 236-240. (10.1038/nature10428) 21964331

[16] Amrein H. 2004 Pheromone perception and behavior in *Drosophila*. Curr. Opin. Neurobiol. **14**, 435-442. (10.1016/j.conb.2004.07.008) 15321064

[17] Ferveur J-F, Sureau G. 1996 Simultaneous influence on male courtship of stimulatory and inhibitory pheromones produced by live sex-mosaic *Drosophila melanogaster*. Proc. R. Soc. Lond. B **263**, 967-973. (10.1098/rspb.1996.0143) 8805834

[18] Pikielny CW. 2012 Sexy DEG/ENaC channels involved in gustatory detection of fruit fly pheromones. Sci. Signal. **5**, pe48. (10.1126/scisignal.2003555) 23131844

[19] Tauber E, Eberl DF. 2001 Song production in auditory mutants of *Drosophila*: the role of sensory feedback. J. Comp. Physiol. A **187**, 341-348. (10.1007/s003590100206) 11529478

[20] Hall JC. 1994 The mating of a fly. Science **264**, 1702-1714. (10.1126/science.8209251) 8209251

[21] Spieth HT. 1974 Courtship behavior in *Drosophila*. Annu. Rev. Entomol. **19**, 385-405. (10.1146/annurev.en.19.010174.002125) 4205689

[22] Yamamoto D, Koganezawa M. 2013 Genes and circuits of courtship behaviour in *Drosophila* males. Nat. Rev. Neurosci. **14**, 681-692. (10.1038/nrn3567) 24052176

[23] Pavlou HJ, Goodwin SF. 2013 Courtship behavior in *Drosophila melanogaster*: towards a ‘courtship connectome’. Curr. Opin. Neurobiol. **23**, 76-83. (10.1016/j.conb.2012.09.002) 23021897 PMC3563961

[24] Dickson BJ. 2008 Wired for sex: the neurobiology of *Drosophila* mating decisions. Science **322**, 904-909. (10.1126/science.1159276) 18988843

[25] Hall JC. 1978 Courtship among males due to a male-sterile mutation in *Drosophila melanogaster*. Behav. Genet. **8**, 125-141. (10.1007/BF01066870) 99136

[26] Villella A, Gailey DA, Berwald B, Ohshima S, Barnes PT, Hall JC. 1997 Extended reproductive roles of the *fruitless* gene in *Drosophila melanogaster* revealed by behavioral analysis of new *fru* mutants. Genetics **147**, 1107-1130. (10.1093/genetics/147.3.1107) 9383056 PMC1208237

[27] Demir E, Dickson BJ. 2005 Fruitless splicing specifies male courtship behavior in *Drosophila*. Cell **121**, 785-794. (10.1016/j.cell.2005.04.027) 15935764

[28] Ryner LC, Goodwin SF, Castrillon DH, Anand A, Villella A, Baker BS, Hall JC, Taylor BJ, Wasserman SA. 1996 Control of male sexual behavior and sexual orientation in *Drosophila* by the *fruitless* gene. Cell **87**, 1079-1089. (10.1016/S0092-8674(00)81802-4) 8978612

[29] Miyamoto T, Amrein H. 2008 Suppression of male courtship by a *Drosophila* pheromone receptor. Nat. Neurosci. **11**, 874-876. (10.1038/nn.2161) 18641642 PMC5655991

[30] Moon SJ, Lee Y, Jiao Y, Montell C. 2009 A *Drosophila* gustatory receptor essential for aversive taste and inhibiting male-to-male courtship. Curr. Biol. **19**, 1623-1627. (10.1016/j.cub.2009.07.061) 19765987 PMC2762023

[31] Lu B, Lamora A, Sun Y, Welsh MJ, Ben-Shahar Y. 2012 ppk23-dependent chemosensory functions contribute to courtship behavior in *Drosophila melanogaster*. PLoS Genet. **8**, e1002587. (10.1371/journal.pgen.1002587) 22438833 PMC3305452

[32] Thistle R, Cameron P, Ghorayshi A, Dennison L, Scott K. 2012 Contact chemoreceptors mediate male-male repulsion and male-female attraction during *Drosophila* courtship. Cell **149**, 1140-1151. (10.1016/j.cell.2012.03.045) 22632976 PMC3365544

[33] Chen S-L et al. 2022 WAKE-mediated modulation of cVA perception via a hierarchical neuro-endocrine axis in *Drosophila* male-male courtship behaviour. Nat. Commun. **13**, 2518. (10.1038/s41467-022-30165-2) 35523813 PMC9076693

[34] Tanaka R, Higuchi T, Kohatsu S, Sato K, Yamamoto D. 2017 Optogenetic activation of the *fruitless*-labeled circuitry in *Drosophila subobscura* males induces mating motor acts. J. Neurosci. **37**, 11 662-11 674. (10.1523/JNEUROSCI.1943-17.2017) 29109241 PMC6705751

[35] Wang K, Guo Y, Wang F, Wang Z. 2011 *Drosophila* TRPA channel painless inhibits male–male courtship behavior through modulating olfactory sensation. PLoS ONE **6**, e25890. (10.1371/journal.pone.0025890) 22073144 PMC3206795

[36] Liu T, Dartevelle L, Yuan C, Wei H, Wang Y, Ferveur J-F, Guo A. 2008 Increased dopamine level enhances male–male courtship in *Drosophila*. J. Neurosci. **28**, 5539-5546. (10.1523/JNEUROSCI.5290-07.2008) 18495888 PMC6670629

[37] Chen B, Liu H, Ren J, Guo A. 2012 Mutation of *Drosophila* dopamine receptor DopR leads to male–male courtship behavior. Biochem. Biophys. Res. Commun. **423**, 557-563. (10.1016/j.bbrc.2012.06.003) 22683328

[38] Zhang SD, Odenwald WF. 1995 Misexpression of the white (w) gene triggers male-male courtship in Drosophila. Proc. Natl Acad. Sci. USA **92**, 5525-5529. (10.1073/pnas.92.12.5525) 7777542 PMC41728

[39] Yin Hing AL, Carlson JR. 1996 Male-male courtship behavior induced by ectopic expression of the *Drosophila* white gene: role of sensory function and age. J. Neurobiol. **30**, 454-464. (10.1002/(SICI)1097-4695(199608)30:4<454::AID-NEU2>3.0.CO;2-2) 8844509

[40] Iyengar A, Imoehl J, Ueda A, Nirschl J, Wu C-F. 2012 Automated quantification of locomotion, social interaction, and mate preference in *Drosophila* mutants. J. Neurogenet. **26**, 306-316. (10.3109/01677063.2012.729626) 23106154 PMC3613147

[41] Ito H et al. 1996 Sexual orientation in *Drosophila* is altered by the satori mutation in the sex-determination gene fruitless that encodes a zinc finger protein with a BTB domain. Proc. Natl Acad. Sci. USA **93**, 9687-9692. (10.1073/pnas.93.18.9687) 8790392 PMC38490

[42] Bloomquist BT, Shortridge RD, Schneuwly S, Perdew M, Montell C, Steller H, Rubin G, Pak WL. 1988 Isolation of a putative phospholipase c gene of drosophila, *norpA*, and its role in phototransduction. Cell **54**, 723-733. (10.1016/S0092-8674(88)80017-5) 2457447

[43] Yamaguchi S, Wolf R, Desplan C, Heisenberg M. 2008 Motion vision is independent of color in *Drosophila*. Proc. Natl Acad. Sci. USA **105**, 4910-4915. (10.1073/pnas.0711484105) 18353989 PMC2290790

[44] Heisenberg M, Wolf R. 1984 Vision in Drosophila: genetics of microbehavior. Berlin, Germany: Springer-Verlag.

[45] Minke B, Wu C-F, Pak W. 1975 Isolation of light-induced response of the central retinula cells from the electroretinogram of *Drosophila*. J. Comp. Physiol. **98**, 345-355. (10.1007/BF00709805)

[46] Reed SC, Reed EW. 1950 Natural selection in laboratory populations of *Drosophila*. II. Competition between a white-eye gene and its wild type allele. Evolution **4**, 34-42.

[47] Krstic D, Boll W, Noll M. 2013 Influence of the *white* locus on the courtship behavior of *Drosophila* males. PLoS ONE **8**, e77904. (10.1371/journal.pone.0077904) 24205022 PMC3813745

[48] Connolly K, Burnet B, Sewell D. 1969 Selective mating and eye pigmentation: an analysis of the visual component in the courtship behavior of *Drosophila melanogaster*. Evolution **23**, 548-559. (10.2307/2406852) 28562876

[49] Phillips JP, Forrest HS. 1980 Ommochromes and pteridines. London, UK: Academic Press.

[50] Lilly M, Carlson J. 1990 smellblind: a gene required for *Drosophila* olfaction. Genetics **124**, 293-302. (10.1093/genetics/124.2.293) 2106470 PMC1203922

[51] Larsson MC, Domingos AI, Jones WD, Chiappe ME, Amrein H, Vosshall LB. 2004 Or83b encodes a broadly expressed odorant receptor essential for *Drosophila* olfaction. Neuron **43**, 703-714. (10.1016/j.neuron.2004.08.019) 15339651

[52] Sharma R. 1977 Light-dependent homosexual activity in males of a mutant of *Drosophila melanogaster*. Experientia **33**, 171-173. 10.1007/BF02124048 403089

[53] Michael PR, Johnston DE, Moreno W. 2020 A conversion guide: solar irradiance and lux illuminance. J. Meas. Eng. **8**, 153-166. (10.21595/jme.2020.21667)

[54] Gao S et al. 2008 The neural substrate of spectral preference in *Drosophila*. Neuron **60**, 328-342. (10.1016/j.neuron.2008.08.010) 18957224 PMC2665173

[55] Ribeiro IMA, Drews M, Bahl A, Machacek C, Borst A, Dickson BJ. 2018 Visual projection neurons mediating directed courtship in *Drosophila*. Cell **174**, 607-621.e18. (10.1016/j.cell.2018.06.020) 30033367

[56] Frankel A, Brousseau G. 1968 Drosophila medium that does not require dried yeast. Drosophila Information Service **43**, 184.

[57] Kaas GA et al. 2016 Lithium-responsive seizure-like hyperexcitability is caused by a mutation in the *Drosophila* voltage-gated sodium channel gene *paralytic*. eNeuro **3**, e0221-16.2016. (10.1523/ENEURO.0221-16.2016) PMC5103163 27844061

[58] Wu CF, Wong F. 1977 Frequency characteristics in the visual system of *Drosophila*: genetic dissection of electroretinogram components. J. Gen. Physiol. **69**, 705-724. (10.1085/jgp.69.6.705) 894240 PMC2215342

[59] Joiner M-LA, Wu C-F. 2004 Nervous system function for the testis RNA-binding protein boule in *Drosophila*. J. Neurogenet. **18**, 341-363. (10.1080/01677060490477435) 15370196

[60] Iyengar A, Wu C-F. 2014 Flight and seizure motor patterns in *Drosophila* mutants: simultaneous acoustic and electrophysiological recordings of wing beats and flight muscle activity. J. Neurogenet. **28**, 316-328. (10.3109/01677063.2014.957827) 25159538 PMC5555410

[61] Ueda A, Berg A, Khan T, Ruzicka M, Li S, Cramer E, Iyengar A, Wu C-F. 2023 Intense light unleashes male–male courtship behaviour in wild-type *Drosophila*. Figshare. (10.6084/m9.figshare.c.6729639) PMC10353890 37463658

